# Whole-Genome Sequencing of 100 Genomes Identifies a Distinctive Genetic Susceptibility Profile of Qatari Patients with Hypertension

**DOI:** 10.3390/jpm12050722

**Published:** 2022-04-29

**Authors:** Alsamman M. Alsamman, Hakeem Almabrazi, Hatem Zayed

**Affiliations:** 1 Department of Genome Mapping, Agricultural Genetic Engineering Research Institute, Agricultural Research Center, Giza P.O. Box 12619, Egypt; smahmoud@ageri.sci.eg; 2 Research Branch, Sidra Medicine, Doha P.O. Box 26999, Qatar; hakeem.almabrazi@gmail.com; 3 Department of Biomedical Sciences, College of Health Sciences, QU Health, Qatar University, Doha P.O. Box 2713, Qatar

**Keywords:** genome-wide association, GWAS, hypertension, genomics, whole-genome sequencing, genomic biomarkers, SNPs, precision medicine, Qatar Biobank, QBB, Qatar

## Abstract

Essential hypertension (EH) is a leading risk condition for cardiovascular and renal complications. While multiple genes are associated with EH, little is known about its genetic etiology. Therefore, this study aimed to screen for variants that are associated with EH in 100 hypertensive/100 control patients comprising Qatari individuals using GWASs of whole-genome sequencing and compare these findings with genetic data obtained from more than 10,000 published peer-reviewed studies on EH. The GWAS analysis performed with 21,096 SNPs revealed 38 SNPs with a significant ≥4 log-*p* value association with EH. The two highest EH-associated SNPs (rs921932379 and rs113688672) revealed a significance score of ≥5 log-*p* value. These SNPs are located within the inter-genic region of *GMPS-SETP14* and *ISCA1P6-AC012451.1*, respectively. Text mining yielded 3748 genes and 3078 SNPs, where 51 genes and 24 SNPs were mentioned in more than 30 and 10 different articles, respectively. Comparing our GWAS results to previously published articles revealed 194 that are unique to our patient cohort; of these, 13 genes that have 26 SNPs are the most significant with ≥4 log-*p* value. Of these genes, *C2orf47-SPATS2L* contains nine EH-associated SNPs. Most of EH-associated genes are related to ion gate channel activity and cardiac conduction. The disease–gene analysis revealed that a large number of EH-associated genes are associated with a variety of cardiovascular disorders. The clustering analysis using EH-associated SNPs across different ethnic groups showed high frequency for the minor allele in different ethnic groups, including Africans, East Asians, and South Asians. The combination of GWAS and text mining helped in identifying the unique genetic susceptibility profile of Qatari patients with EH. To our knowledge, this is the first small study that searched for genetic factors associated with EH in Qatari patients.

## 1. Introduction

Cardiovascular disorders cause about 17 million deaths worldwide, with about one-third of these being due to hypertension complications [1]. Essential hypertension (EH) is a chronic and age-related disorder that frequently causes cardiovascular and renal risks. EH affects 25–35% of the adult population in both developed and developing countries, leading to stroke and cardiovascular disorders. Of these, up to 60–70% are in their mid-sixties [2,3]. Several factors, such as the large arteries, endocrine factors, central nervous system, and microcirculation, are involved. The correlation between these factors varies with age and reflects the heterogeneous pattern of hemodynamic changes [2]. A data survey of the global burden of disease showed that in 2015, 7.8 million deaths were related to a systolic blood pressure of ≥140 mmHg, which is the current clinical threshold for identifying hypertension [4].

Both genetic and environmental factors are involved in the development of EH symptoms [5,6]. Although several research efforts have been made, the genetic etiology of EH remains partly unknown. EH is a polygenic condition that results from the inheritance of a variety of risk genes, each with a small influence according to sex, age, race, or lifestyle [2,7]. Population-based studies have reported that hypertension runs in approximately 20% of families, which might be related to genetic factors and/or dietary, lifestyle, and activity levels [8]. In twin studies, this risk was up to 60%, where dizygotic twins showed a lower association than monozygotic twins [2,9].

The hereditary contribution to hypertension variations is estimated to be around 30%, but the genetic history of critical hypertension is ambiguous and poorly understood [10]. Several studies have associated dozens of genes with hypertension. However, genes linked to the function of the kidneys that are involved in fluid and electrolyte balance control are the most significant determinants of blood pressure [11]. These genes include aldosterone synthase genes *CYP11B1* and *CYP11B2* [12], *HSD11B2* [13] *eNOS* [14], and others. Genetic variants play a significant role in the malfunctioning of such genes, resulting in unpredictability in the primary biological pathways controlling blood pressure. Furthermore, recent research indicates that epigenetic mechanisms, such as DNA methylation, post-translational histone modifications, and microRNAs, play a significant role in the molecular dynamics underlying EH etiopathogenesis. Some of these modifications affect blood pressure and thus lead to a variety of cardiovascular disorders [12].

Single-nucleotide polymorphisms (SNPs) are one of the most common genetic variations in humans, and they are the most common and functionally important source of evolution [15]. Rapid advances in sequencing technologies permit affordable, reliable population SNP genotyping. These technologies offer the ability to screen thousands of SNPs that could be used as biomarkers to identify hypertension susceptibility loci through genome-wide association studies (GWAS) [7,16]. GWASs apply a statistical inference approach, applying estimation and hypothesis testing on the correlation between a trait and variants such as SNPs [17]. The rate of identification of validated SNPs in GWASs associated with blood pressure has recently increased exponentially [18,19]. GWASs enabled medical researchers to identify several loci that may contain causative variants or genes that lead to overall EH susceptibility at a single locus [20]. Furthermore, the ongoing global development of national biobanks has provided researchers with sufficient bio-specimens for well-established cohort studies to explore genome-wide significant associations for EH in specific ethnic groups [21].

Drawing inferences from what is already known is an important process in creating knowledge [22]. In this context, we can draw more insightful conclusions by analyzing the available information about EH. Text mining is a method of data mining in which a large volume of structured and unstructured text data are processed to generate potentially valuable information [23]. Due to the significant expansion of medical knowledge, text mining is being used to enhance medical analysis and provide more comprehensive techniques for data processing. It is a rich resource for the acquisition of knowledge from the current research literature. The only downside is that it requires a high level of data filtration and manipulation skills [24,25]. It enables researchers to classify various database resources, speed up the structuring of information derived from unstructured data, and gain deeper insights. A text mining approach was used to organize and evaluate published scientific literatures on cancer [26], diabetes [27], and schizophrenia [28]. Some reports used text mining to classify biomedical literatures in hypertension and to identify useful treatments using linguistic techniques [29].

We conducted a GWAS on 100 Qatari patients with hypertension and 100 normal Qatar individuals. Additionally, we performed text-mining analysis to identify the unique variants that predispose Qataris to hypertension.

## 2. Methodology

### 2.1. SNPs Associated with EH in Qatari Population

#### 2.1.1. Study Design and Statistical Analysis

**Figure 1:**
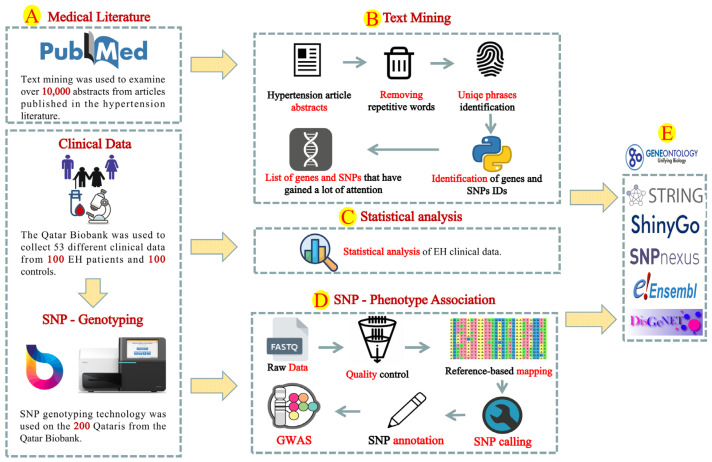
A flowchart representing the genetic resources and bioinformatics methods used in the present research. Thousands of hypertension-related articles were reviewed, as well as the SNP genotyping of 200 Qataris with high blood pressure (**A**) using text mining (**B**), statistical analysis (**C**), and GWAS (**D**). The text mining analysis included eliminating redundant words, recognizing unique phrases, detecting genes and SNP expressions, and categorizing these genes and SNPs based on their prevalence and redundancy (**B**). Fifty-three different clinical data of the studied patients were retrieved from QBB and used for statistical analysis (**C**). The SNP genotyping data were analyzed using a quality control method before being aligned/mapped to the human genome, where discovered SNPs were annotated and their association with high blood pressure was investigated using GWAS analysis (**D**). The details obtained from text mining and GWAS analysis was analyzed using gene ontology and enrichment analyses (**E**).

Figure 1 is a schematic representation of the methodology section. The data of the cohort studied were acquired from the Qatar Biobank (QBB), which comprised 100 patients with EH and 100 normal controls. All participants were Qatari [30] and were sequenced through the national Qatar genome project [31] (Figure 1A). The eligibility criteria for hypertensive subjects were: (1) age ≥ 35–70; (2) systolic blood pressure (SBP) ≥ 140 mmHg and/or diastolic blood pressure (DBP) ≥ 90 mmHg; (3) absence of secondary causes of hypertension based on comprehensive biochemical and clinical studies; and (5) absence of pharmacological therapy for hypertension. Fifty-three different associated data of both the control and patients were retrieved from QBB and used for statistical analysis (Supplementary S1). These data include clinical parameters, such as age, blood pressure factors, body mass index, sodium, potassium, chloride, bicarbonate, urea, creatine kinase, creatinine, glucose, total protein, albumin, cholesterol, triglyceride, calcium, phosphorus, iron, magnesium, Fibrinogen, vitamin B12, insulin, and others. The statistical analyses, which included principal component analysis and correlation, were carried out in R, and missing values were imputed using the “mice” package with the predictive mean matching (PMM) method [32].

#### 2.1.2. SNP Genotyping and Computational Analysis

DNA was isolated from 5 mL of blood samples using the Puregene DNA extraction kit (Gentra Systems, Minneapolis, MN, USA) according to the manufacturer’s instructions. DNA quantification was performed using Fluorometer Qubit 2.0 (Invitrogen, Carlsbad, CA, USA). Whole-genome sequencing (WGS) was conducted by QBB on DNA samples from 200 participants. Illumina HiSeq X Ten sequencers were used for WGS analysis, where raw data were processed using bioinformatics pipelines (Figure 1C). FastQ data were converted to paired-end FASTQ format using bcl2fastq conversion tool. FastQC software was used to assess the raw data quality. The human genome GRCh37 version was used as a reference for data passing quality control using Burrow-Wheeler Aligner (BWA) aligner (v7.12). SNP calling was performed using HaplotypeCaller provided by Genome Analysis Toolkit (GATK v3.3) [33]. The SNP effect and annotation were categorized using SnpEff (v4.1) [34]. SNP was discarded from analysis if the genotyping call rate was <95%, the minor allele frequency (MAF) was <1%, the heritability error rate was >1%, or the Hardy–Weinberg equilibrium was *p* < 10^−6^. Filtered SNPs were used for further analysis. Fisher’s exact test [35] was conducted to determine the statistical significance of allele frequency differences between case and control groups (Figure 1C).

### 2.2. Exploring Published Hypertension Literature

The available hypertension reports were explored in order to obtain more context on the prior information of the genetic mechanism of hypertension (Figure 1A,B). PubMed–NCBI was used to retrieve all abstracts of the scientific articles that reported hypertension-associated genes and SNPs (Figure 1A,B). The query of “hypertension genes” was used to download all abstracts of medical articles published up to “Tue, 24 November 2020 05:35:29”. The text analysis included “10,001” different articles. The data mining was conducted through Python programming language (Supplementary S2). Common English phrases and word redundancy were removed. A list of human gene terminology was prepared using human genome (GRCh38 version) obtained from the NCBI database. The SNP reference numbers (RSs) were extracted using regular expression and string-searching algorithms (Supplementary S2).

### 2.3. Gene and SNP Enrichment Analysis

The collected hypertension-associated genes from the GWAS and text mining analyses were used for gene annotation and enrichment analysis. Comprehensive computational analysis was conducted using several bioinformatics tools (Figure 1D). Gene enrichment analysis was conducted using ShinyGo [36] and uniport database [37]. SNPnexus and Ensembl [38] platforms were used to collect more information about hypertension-associated SNPs retrieved from text mining and GWAS analyses, including allele frequencies in different ethnic populations, gene annotation, and pathway analysis. We analyzed protein–protein interactions (PPIs) using the STRING database and Cytoscape software, and gene names were used as queries to extract information from gene ontology databases in STRING platform [39] (Figure 1D). Text mining and GWAS results are represented using Circos software [40], R-ggplot2 [41], and GeneSyno [25] according to the human genomic data. Online Clustvis tool was used to perform statistical clustering [42]. The disease gene analysis was conducted using disease genomics of DisGeNET [43].

## 3. Results

### 3.1. Statistical Analysis

**Figure 2:**
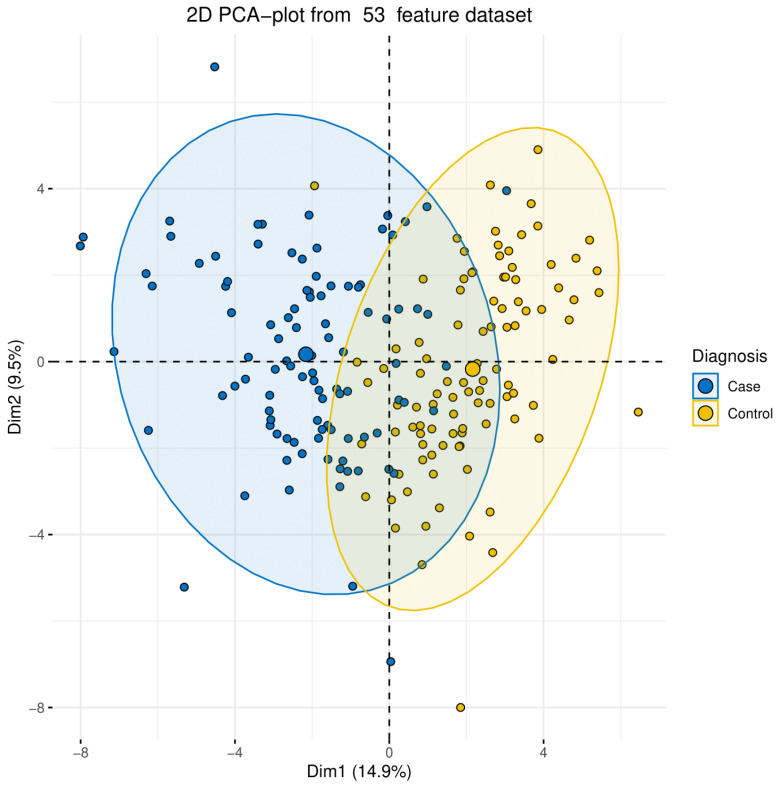
The principal component analysis of hypertension data. The PCA analysis of the 200 Qataris clustering based on 53 different clinical parameters, including blood pressure measurements.

**Figure 3:**
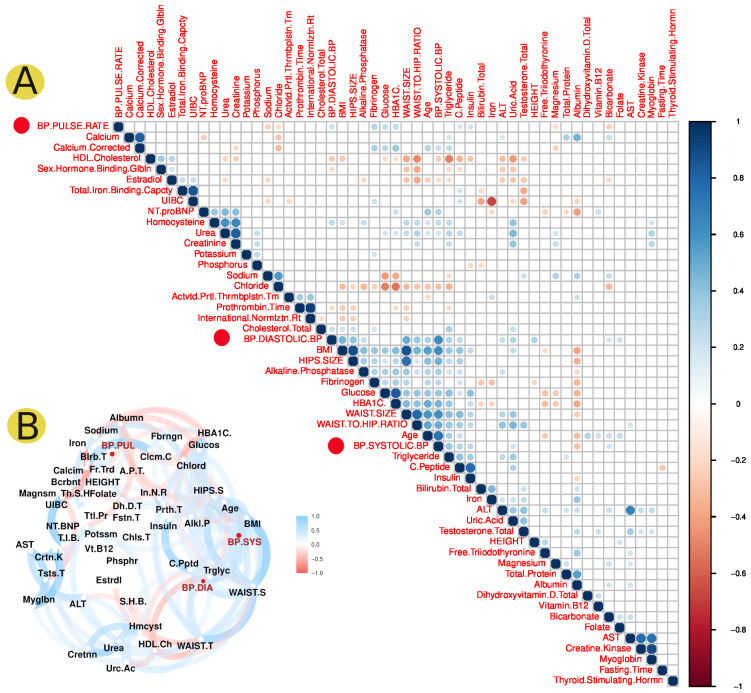
The statistical correlation analysis of hypertension data. The correlation analysis of different clinical parameters from the 200 Qataris obtained from QBB, with blood pressure parameters highlighted in red circles. (**A**) Graph of a correlation matrix showing positive and negative correlations with significance indicated by *p* values less than 0.01. (**B**) A correlation network graph displaying the statistical interaction between various clinical parameters, including blood pressure factors in red.

Principle component analysis (PCA) is a common analysis used for sample classification and clustering. It reduces the dimensionality of the investigated dataset, improving interpretability while minimizing information loss. We used this type of analysis to group the studied individuals (100 case and 100 controls) based on several clinical data parameters that did not include the medical diagnosis. The PCA analysis showed that the clinical parameters obtained in this study were useful in highlighting the main characteristics that distinguish hypertension. The PCA analysis revealed that the patients studied were clustered based on their hypertension diagnosis (Figure 2). The statistical correlation of clinical data revealed a significant correlation between blood pressure factors such as systolic blood pressure, diastolic blood pressure, blood pressure/pulse rate, and several clinical parameters (Figure 3A,B). For instance, systolic and diastolic blood pressure showed a significant positive correlation with body mass index, Alkaline phosphatase, and C peptide, while blood pressure/pulse rate was positively correlated with C peptide, insulin, Fibrinogen, and HbA1C (Figure 3A).

### 3.2. SNPs Associated with EH in Qatari Population

**Figure 4:**
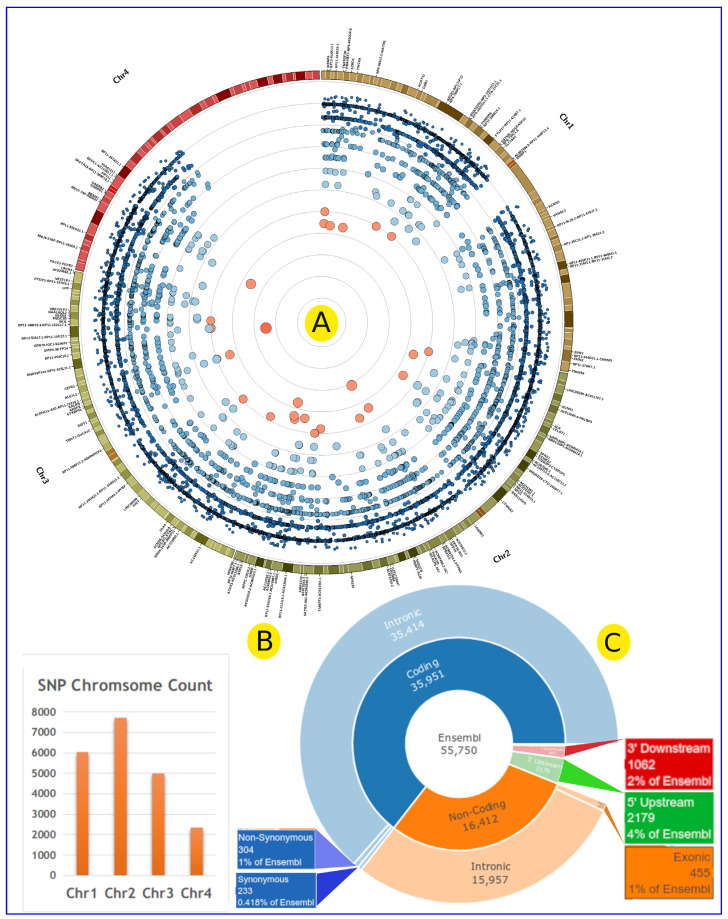
The chromosomal distribution of the identified SNPs and their association scores with EH. The colored small circles represent SNPs’ location on the human genome and their hypertension association score (−log10 of *p* value) according to the Fisher’s exact test. Blue and small circles represent non-significant SNPs, while large and red circles represent significant SNPs (**A**). The number of SNPs according to human chromosome (**B**). Predicted functional consequences. SNPs are classified into several groups based on their location in and effect on gene structures, which include coding and non-coding regions according to different databases (**C**).

In this study, we investigated the association between hypertension and 21,096 SNPs in 1503 genes across the human genome that are specific to our patient cohort (100 cases vs. 100 normal controls) (Figure 4A). Our analysis revealed hypertension-associated genes in four human chromosomes (Chr 1, 2, 3, and 4) that are specific to our 100 patients. Chr2 had the highest number of SNPs, followed by chromosome Chr1. The SNP density for 1 Mbp was 30, 36, 29, and 28 for Chr1, 2, 3, and 4, respectively (Figure 4A). The estimated functional consequences of the distribution indicate that the used SNPs are mainly located within coding regions (Figure 4C). Most of the SNPs are located in genes (12,913), while 8995 SNPs are upstream or downstream genes with a distance varying from 5 bp to 21 Mbp. Genes such as *DPP10* (119 SNPs), *AC007319.1* (105 SNPs), *RN7SKP61* (101 SNPs), and *RN7SL647P* (92 SNPs) acquired the highest number of SNPs (Supplementary S3).

**Figure 5:**
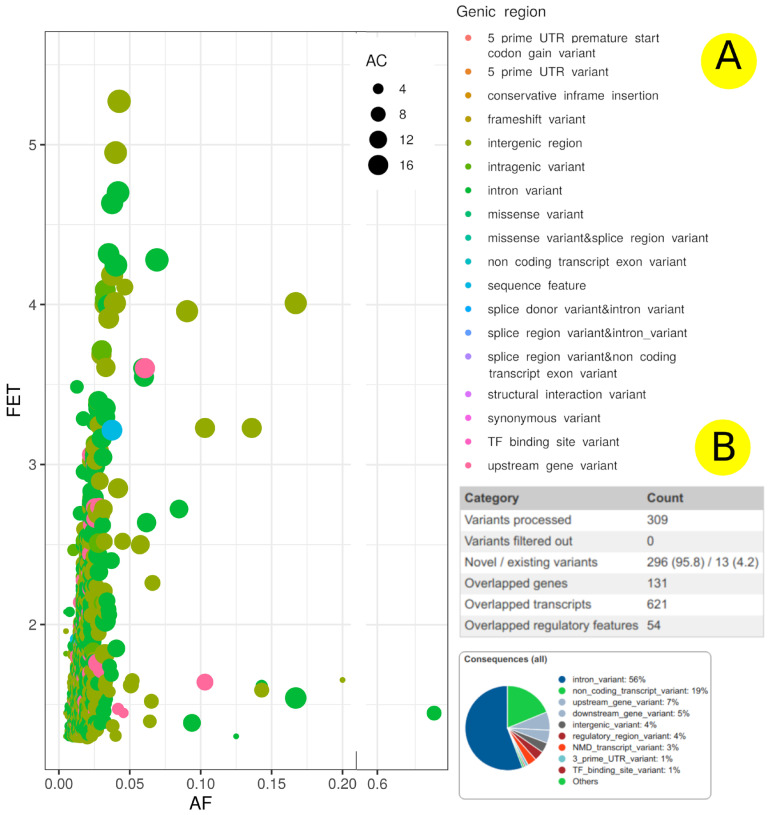
The genic structure of SNPs associated with EH. (**A**) The allele frequency vs. FET scores -log10 of the studied SNP variants. Allele frequency of SNP variants (*x*-axis) vs. FET scores -log10 (*y*-axis), the SNP genic region (color) and the maximum allele count in studied population (size). (**B**) The percentage of SNPs distribution across gene structure.

**Figure 6:**
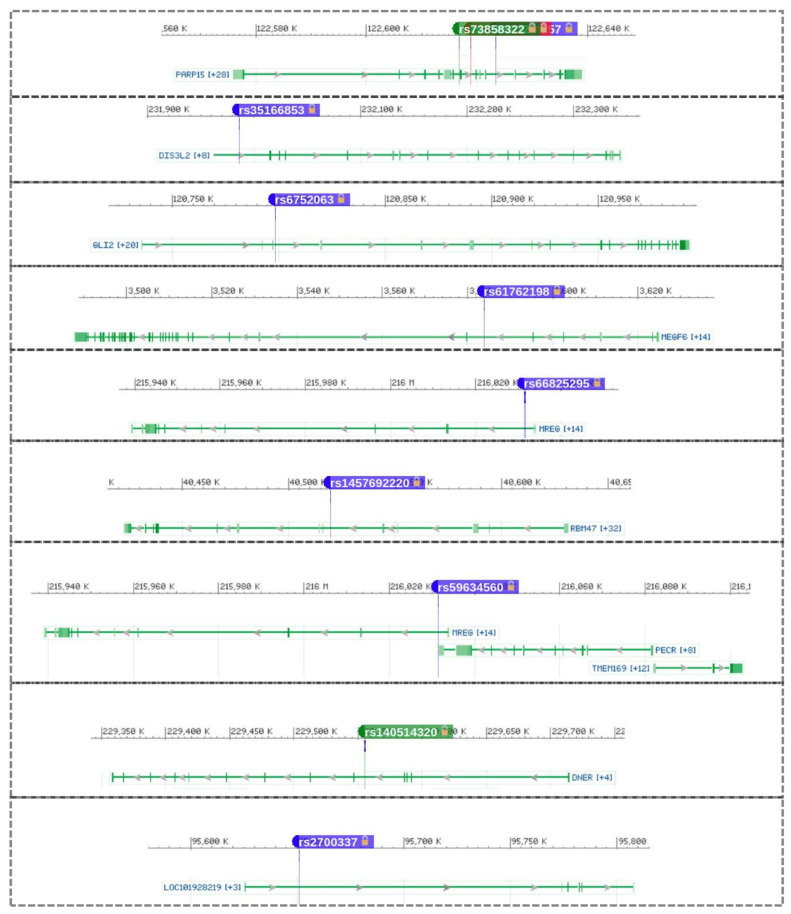
Graphical representation of the location of some SNPs linked to hypertension. The physical location of several SNPs discovered to be associated with hypertension using GWAS analysis.

The correlations between allele count (AC), Fisher’s exact test (FET), genic region, and allele frequency are plotted in Figure 5. This correlation is important to reveal any potential misinterpretation of the genetic association with EH and visually demonstrate the potential impact of each genetic variation feature on SNP association with EH manifestation. Furthermore, examining the relationship between genetic variation components and EH may reveal previously unknown parameters that could be used in future EH genetic research to increase the number of significant SNPs. Mostly, smaller numbers of SNPs with higher FET have more AC and AF, which could indicate their importance in EH. SNPs with FET ≥ 4 are located in the intergenic region (20 SNPs), intron variant (10 SNPs), intragenic variant (5 SNPs), and upstream gene variant (3 SNPs) (Figure 5, Table 1 and Supplementary S4). The FET analysis revealed 336 SNPs with ≥3 log-*p* value, 38 SNPs with ≥4 log-*p* value, and 2 SNPs (rs921932379 and rs113688672) with ≥5 log-*p* value (located within regions of *GMPS-SETP14* and *ISCA1P6-AC012451.1* genes) (Figure 4, Table 1, and Supplementary S4). These SNPs are near/adjoined to 215 genes (Figure 5 and Figure 6 and Supplementary S5), and the SNPs’ distribution across the gene structure can be seen in Figure 5 and Figure 6.

**Figure 7:**
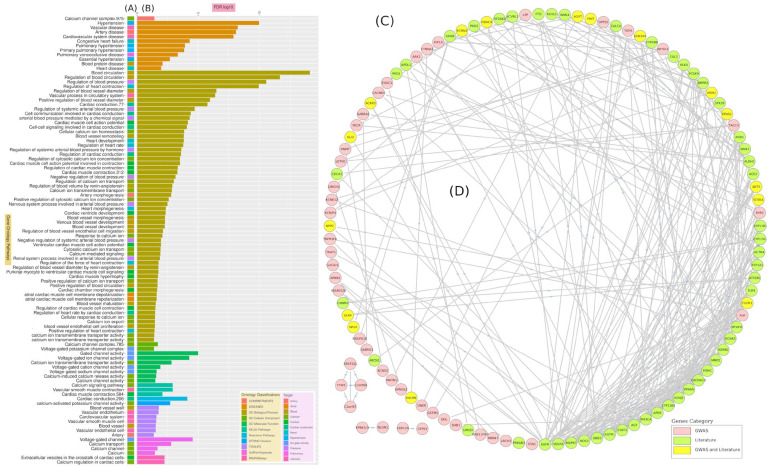
The gene ontology and PPI analysis of highly frequently occurring genes that are associated with EH in both the literature and our GWAS analysis. The most important gene ontology terms associated with EH (**A**) and their ontology class (**B**). The PPI interaction (**C**) of EH-associated genes found through GWAS and literature analysis separately and together; connecting lines (**D**) signify the biological interactions.

We identified three novel SNPs: rs_new-95 (4.70 log-*p* value), rs_new-58 (3.06 log-*p* value), and rs_new-56 (4.11 log-*p* value). These SNPs are located in Chr4:40521676, Chr2:221398860, and Chr2:193355414, respectively (Table 1 and Supplementary S4). A total of seven genes have ten or more SNPs with ≥3 log-*p* value, including *MAIP1* (23 SNPs), SPATS2L (21 SNPs), ULK4 (17 SNPs), *PKN2-AS1* (14 SNPs), *AC092966.1* (11 SNPs), *FHIT* (10 SNPs), and *RNA5SP52* (10 SNPs) genes (Figure 6 and Supplementary S3). The PPI analysis for the genes where significant SNPs are located demonstrated interaction activity across the hypertension-associated proteins. On the other hand, it shows high significance of functional analysis for genes related to ion-gated channel activity and cardiac conduction (Figure 7, Table 1).

**Figure 8:**
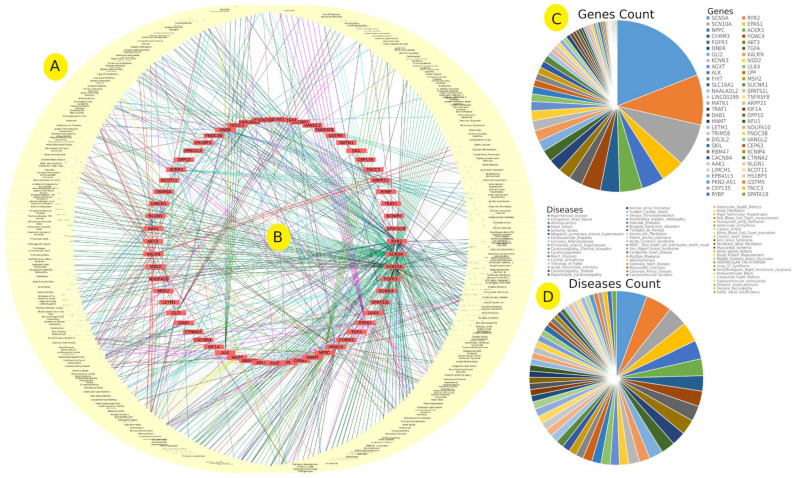
The disease–gene analysis of the cardiovascular disorders (**A**) and genes associated with hypertension (**B**) in the studied Qatari population. The comparison of the presence of various genes (**C**) and disease (**D**).

We explored the correlation between our detected EH-associated SNPs and a variety of cardiovascular disorders. The gene–disease association analysis revealed that 60 EH-associated genes are linked to 270 cardiovascular disorders (Figure 8 and Supplementary S6). The *SCN5A* gene that showed a correlation between rs74947646 and EH with a log-*p* value of 3 is linked to more than 121 cardiovascular disorders (Supplementary S6). The various types of long QT syndrome (LQTS) were highly represented in the disease associated with *SCN5A* variants (Supplementary S6). The *RYR2* gene was linked to 60 different cardiovascular disorders, the majority of which affect the functionality of ventricular hypertrophy. We identified rs1391189881 in the *RYR2* gene with a significant association of a log-*p* value of 3 (Supplementary S4). Furthermore, genes of *SCN10A*, *EPAS1*, *NPPC*, *ACKR3*, *CHRM3*, and *HDAC4* were linked to 51, 28, 27, 27, 23, and 23 cardio disorders, respectively (Figure 8 and Supplementary S4). Both *EPAS1* and *NPPC* have been linked to two SNPs: rs113717961 (3 log-*p* value) and rs115272974 (3 log-*p* value), and rs34189801 (4.316952962 log-*p* value) and rs34553499 (4.316952962 log-*p* value), respectively (Supplementary S4). Hypertensive disease was represented by 20 different EH-associated genes, followed by multiple myeloma (17 genes), congestive heart failure (13 genes), atherosclerosis (13 genes), arteriosclerosis (12 genes), coronary heart disease (11 genes), heart failure (10 genes), myocardial infarction (10 genes), ischemic stroke (10 genes), and coronary artery disease (10 genes) (Figure 8 and Supplementary S4). Idiopathic pulmonary arterial hypertension, pulmonary arterial hypertension, hypertensive nephropathy, and renin-induced hypertension are all examples of EH-like disorders associated with the identified EH genes (Supplementary S4). These disorders were found to be linked to 14 different genes and 16 SNPs (Supplementary S4).

### 3.3. Hypertension-Associated Genes’ Recent Status in the Literature

**Figure 9:**
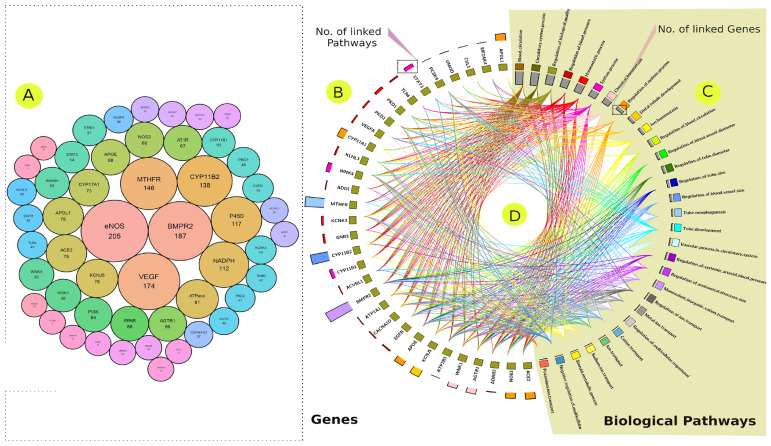
The most frequently mentioned genes in the hypertension literature (**A**,**B**). The size of the circle, as well as the numbers included, represent the number of articles that mentioned a genetic link between these genes and EH disorder (**A**). The most frequently mentioned genes in the hypertension literature and their corresponding biological processes according to the enrichment analysis (**C**). The histogram depicts the number of hypertension publications that listed these genes (**A**,**B**). Biological processes were allocated to hypertension-associated genes (**C**), with gray bars representing the number of genes found within these processes and colored connections representing genes that contribute to these processes. A colored link is shown between the biological process and the corresponding EH-linked genes, with each gene potentially linked to multiple pathways or vice versa (**D**).

We used text mining to screen previously published research articles related to gene associations with EH. Our search yielded 10,001 hypertension-related articles published in the last ten years, which we compared with our GWAS findings and the published data, enabling us to map unique or novel gene/variants that are specific to our cohort. The text mining analysis revealed 3078 SNPs in more than 3748 genes. Of these, 51 genes and 24 SNPs were mentioned in more than 30 and 10 different articles, respectively (Supplementary S7). Among these genes, *eNOS*, *BMPR2*, *VEGF*, *MTHFR*, *CYP11B2*, *P450*, and *NADPH* were mentioned in more than 100 scientific articles (Figure 9 and Supplementary S7). The SNPs of rs1799983, rs2070744 (NOS3 gene), rs699 (AGT gene), and rs5186 (AGTR1 gene) were detected in more than 20 different articles (Supplementary S7). The gene enrichment analysis revealed a high association with functional categories such as the circulatory system process, blood circulation, and regulation of blood pressure (Figure 9). The PPI analysis showed that genes such as *MAPK3*, *NOS3*, *AGTR1*, *DECR1*, *VEGFA*, *GNB3*, *APOE*, *CYP11B2*, *EGFR*, *POMC*, and *STAT3* were highly interactive in pathways that are related to homeostatic processes, nitric-oxide-mediated signal transduction, blood circulation, and regulation of blood pressure (Figure 7).

### 3.4. GWAS and Text Mining Results Comparison

**Figure 10:**
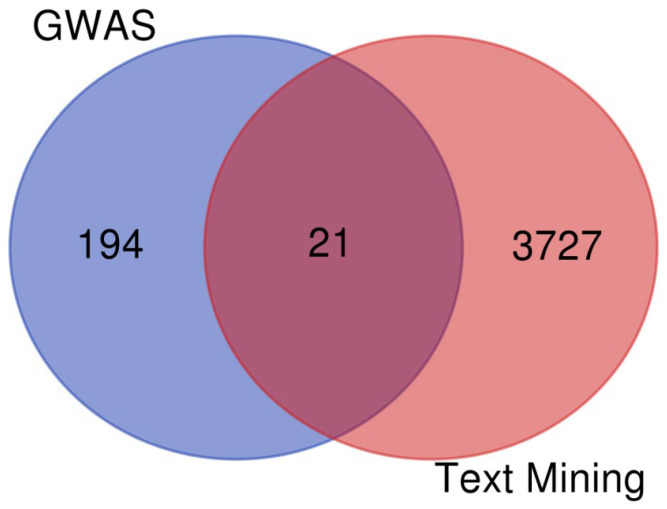
Comparison between the EH-associated genes identified in the GWAS and text mining analysis.

The text mining analysis yielded 3748 genes that are associated with hypertension in the published literature (Supplementary S7). We compared this list of genes with our GWAS hypertension-related genes with SNPs with ≥3 log-*p* value association scores. We identified 21 genes that are common between the GWASs and text mining that contain 50 SNPs significantly associated with hypertension in our studied cohort (Figure 10 and Supplementary S4 and S8). Genes of *ULK4* and *FHIT* have the highest number of hypertension-associated SNPs for genes mentioned in previous studies (Supplementary S5). *ULK4* and *FHIT* genes have 17 and 10 EH-associated SNPs, respectively, while *FNDC3B*, *FHIT*, *NPPC-DIS3L2*, *GLI2*, and *RBM47* include SNPs that have a ≥4 log-*p* value association with hypertension in the studied cohort. On the other hand, 194 are unique to our patient cohort; of these, 13 genes that have 26 SNPs are the most significant with ≥4 log-*p* value. Of these genes, *C2orf47-SPATS2L* contains nine EH-associated SNPs.

### 3.5. Allele Frequency of EH-Associated SNPs across Ethnic Groups

**Figure 11:**
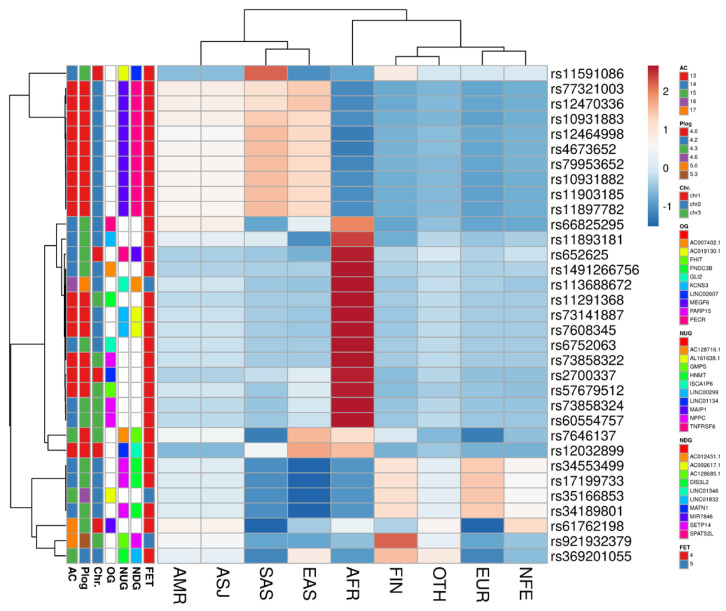
The clustering analysis of hypertension-associated SNPs (≤4 log-*p* value) across different ethnic groups. The SNP identity number (SNPID), maximum allele count (AC), EH association log-*p* value score, chromosome (Chr), overlapped gene (OG), nearest upstream gene (NUG), and nearest downstream gene (NDG).

To study the frequency of the SNPs identified through our GWAS with ≥3 log-*p* value in different ethnic populations, we screened our associated SNPs in EUR, SAS, AFR, AMR, ASJ, EAS, FIN, NFE, and OTH populations (Figure 11 and Supplementary S9). The clustering analysis of SNPs’ frequency across different ethnic groups demonstrated high frequency for the minor alleles, especially in AFR, EAS, and SAS populations. For instance, rs1004840 shows a high-frequency rate of 0.24 for the minor allele (T) in the AFR population compared to the major allele (C:0.76) (Supplementary S9). Additionally, the clustering analysis using EH-associated SNPs revealed some consistency for SNP clustering depending on the *p* values, AC, overlapped gene (OG), nearest upstream gene (NUG), and nearest downstream gene. Furthermore, a high number of EH-SNPs (rs11591086, rs77321003, rs12470336, rs10931883, rs12464998, rs4673652, rs79953652, rs10931882, rs11903185, and rs11897782) have separated the populations of AMR, ASJ, SAS, and EAS with high frequencies for the major allele (Figure 11). These SNPs are located near the *SPATS2L* (nine SNPs) and *MATN1* (one SNP).

## 4. Discussion

Hypertension is a complicated cardiovascular condition that is influenced by various genetic and environmental factors. More basic research and sophisticated tools are needed to understand the missing part of heritability of hypertension. The strength of this study is the combination of a GWAS of 100 hypertensive cases and 100 controls of whole-genome-sequenced individuals and text mining of past reports about the genetic association with hypertension in the past ten years.

We studied 53 different health parameters of both the cases and controls. PCA demonstrated that the clinical parameters obtained in this study were useful in highlighting the main characteristics that distinguish hypertension (Figure 2). PCA demonstrated a good clustering of EH patients and was useful in distinguishing the majority of patients from controls based on the clinical data. A low number of explanations were detected on both axes. On the other hand, this could be attributed to the high complexity of EH and the low precision of clinical parameters alone in detecting EH contributing factors. The correlation analysis of the clinical data revealed a strong association between high blood pressure factors and several clinical parameters (Figure 3). Additionally, clinical parameters related to alkaline phosphatase and C peptide, fibrinogen, HbA1C, iron, homocysteine, and serum ferritin showed a significant association with hypertension (Figure 3).

### 4.1. Genome-Wide Association

The outcome of the GWAS of 100 hypertensive patients and 100 normal controls yielded 21,096 SNPs within 1503 genes that associated with hypertension (Figure 4 and Figure 6). Our study identified two SNPs, rs921932379 and rs113688672, associated with hypertension (≥5 log-*p* value), which are located within the intergenic region of *GMPS*-*SETP14* and *ISCA1P6-AC012451.1* genes, respectively (Table 1, Figure 5 and Figure 6). The association between these loci and hypertension risk has not been reported in the medical literature. GMPS (guanine monophosphate synthesis) is engaged in guanine nucleotide de novo synthesis providing GTP, which is involved in cellular processes that are critical for cell division [44]. GTP is the primary source of cyclic guanosine monophosphate (cGMP), which catalyzes a variety of cardio-protective functions [45]. cGMP signaling stimulation is a possible therapeutic strategy for heart failure [46]. The association between the *AC012451.1* gene (a long non-coding RNA/lincRNA) and hypertension may be due to their role in gene regulation [47]. On the other hand, *SETP14* and *ISCA1P6* are pseudogenes, which are non-functional segments of DNA that resemble functional genes. The link between certain pseudogene genes and heart disease has previously been reported in human and model animal studies and is currently an active area of medical research [48,49,50]. For example, the *HK2P1* pseudogene may contribute to preeclampsia by acting as a competing endogenous RNA for hexokinase 2 and impairing decidualization [48].

We identified three novel SNPs through our GWAS: rs_new-95 (4.70 log-*p* value), rs_new-56 (4.11 log-*p* value), and rs_new-58 (3.06 log-*p* value) (Table 1, Supplementary S4). These are insertion and deletion SNPs found within *RBM47*, *TMEFF2-AC013401.1*, and *AC067956.1*, respectively. The *RBM47* (RNA Binding Motif Protein 47) gene is an RNA-binding protein that controls cell fate decisions and has been suggested to be a tumor suppressor [51]. Rare putative functional hypertension-associated variants in the *RBM47* gene were discovered [52]. These reported rare SNPs could promote hypertension under some environmental factors [53,54]. *TMEFF2* (Transmembrane Protein with EGF-Like and Two Follistatin-Like Domains 2) has two follistatin-like domains that interact directly with TGF-β and thus regulate associated growth factor signaling and, as a result, blood pressure regulation. *TMEFF2* has been linked to hypertension and cardiac hypertrophy, according to previous research [55,56].

Seven genes have more than 10 SNPs with ≥3 log-*p* value (Supplementary S2). Among these genes, Spermatogenesis-Associated Serine-Rich 2 (*SPATS2L*) revealed 21 hypertension-associated SNPs, of which 9 SNPs were ≥4 log-*p* value (Table 1). *SPATS2L* has a well-known role in the manifestation of some human disorders such as asthma [57] and hepatocellular carcinoma [58]. Recently, some reports have defined a clear link between *SPATS2L* and atrial fibrillation, which is an abnormal and sometimes rapid heart rate that occurs when the two upper chambers of the heart have unstable electrical signals [59]. Additionally, the Unc-51-Like Kinase 4 (*ULK4*) gene revealed 17 SNPs with ≥3 log-*p* value (Figure 4 and Figure 6). Several reports have mentioned the association between the *ULK4* gene and heart disorders, including acute aortic dissections [60] and hypertension [61]. *ULK4* has a well-known role in neuronal growth and tyrosine kinase activity [62]. Among genes with a high number of hypertension-associated SNPs, the PKN2-AS1 lncRNA region revealed 14 SNPs. The Protein Kinase N2 (*PKN2*) gene plays a role in the regulation of cell cycle progression, cell migration, actin cytoskeleton assembly, and tumor cell invasion. Several blood-pressure-associated loci were reported in *PKN2* [63]. Additionally, the Fragile Histidine Triad Diadenosine Triphosphatase (*FHIT*) gene revealed 10 hypertension-associated SNPs with ≥3 log-*p* value, 1 of which was an SNP (rs57679512) with ≤4 log-*p* value. *FHIT* was a hot point for cancer research for decades [64]. Recently, *FHIT* has gained great interest as a key factor for pulmonary arterial hypertension, where its reductions were associated with endothelial and smooth muscle cell dysfunction [65].

The PPI analysis revealed interaction activity across the previously hypertension-associated genes; this could be due to their unknown interaction on the cellular level and possible unseen links. On the other hand, the PPI shows high significance of functional analysis for genes related to ion-gated channel activity and cardiac conduction (Figure 7). This is highly accepted as most of genes are correlated with signal transduction activity and muscle proliferation and regulation. The relationship between hypertension and cell voltage-gated ion channels, especially in arteries, is well-known where this cellular process is related to multifactorial mechanisms [66].

#### Disease–Gene Relationship

The disease–gene relationship analysis reveals that cardiovascular disorders, including hypertension, are more closely associated with genetic variants located in a specific several hub genes (Figure 8B,C). These genes are linked to a number of biological pathways that are important in regulating cellular processes, such as signal transduction and cell development. We found that 61 of the detected EH-associated genes are linked to 270 cardiovascular disorders (Figure 8 and Supplementary S6). *SCN5A* was linked to 121 cardiovascular disorders (Figure 5 and Supplementary S6). Several studies have examined the relationship between *SCN5A* and a variety of cardiac disorders [67]. Dilated cardiomyopathy, cardiac conduction disease, and sick sinus syndrome are all cardiac conditions linked to variants in *SCN5A* [68,69]. The alpha subunit of the main cardiac sodium channel Nav1.5 is encoded by *SCN5A*, which regulates cardiac electrophysiological function [69]. Our study revealed that rs74947646 (located in *SCN5A-SCN10A*) is associated with EH (log-*p* value ≥ 3) (Supplementary S4). Similarly, *RYR2*, which contains rs1391189881 (log-*p* value ≥ 3), has been linked to 60 different cardiovascular disorders (Supplementary S4 and S6). The *RYR2* gene codes for a protein known as ryanodine receptor 2, which forms channels within cells that transport positively charged calcium ions. These channels are important in the heart contraction process [70]. The identified EH-associated *SCN10A* is known for its influence on cardiac conduction by controlling the activity of sodium channels, which are required for electrical signal transmission in cells [71]. *SCN10A* has been linked to 51 cardiovascular disorders and shares an EH-associated SNP with *SCN5A* according to our GWAS analysis (rs1391189881 with log-*p* value ≥ 3) (Supplementary S4 and S6). Interestingly, some known SCN10A variants have been reported to modulate cardiac *SCN5A* expression, influencing cardiac physiology and factors that predispose individuals to arrhythmia [71]. Furthermore, *EPAS1* has a high number of cardio disorders and is linked to two different EH-SNPs (Supplementary S4). EPAS1 induces adrenomedullin hormone expression and plays an important role in cardiac myocyte inflammation [72,73]. These results suggest that our identified SNPs associated with EH are likely to be correlated with EH. In addition, we discovered that the majority of EH-associated genes are strongly linked to cardiovascular disorders via 14 different genes and 16 SNPs (Supplementary S4).

### 4.2. Text Mining in the Hypertension Literature

The text mining analysis was used to explore the previously reported genes associated with hypertension. The hypertension articles published in the last decade revealed a small number of highly redundant hypertension-associated genes and SNPs. We identified 51 genes; each of them was mentioned at least in 30 articles. In addition, 24 SNPs were captured, each of which was mentioned in at least 10 research articles (Figure 9 and Supplementary S7). This could lend credence to the known complexities of the key factors that govern such a condition [74]. Most of these genes are correlated with blood circulation, while others are related to ion transportation, the metamorphosis of coronary arteries, and blood vessels (Figure 9C). Among these genes, eNOS/NOS3, *BMPR2*, *VEGF*, *MTHFR*, *CYP11B2*, *P450*, and *NADPH* were highly redundant (Figure 9B). Because of their well-known and mostly direct link to hypertension molecular mechanisms, these genes are frequently mentioned in the hypertension-related studies.

The PPI network was constructed for the genes identified with text mining to visualize the EH-associated genes. Interaction activity revealed a collection of genes forming a hot spot of biological activity (Figure 7). These highly interactive genes are mostly related to the homeostatic process, nitric-oxide-mediated signal transduction, blood circulation, and regulation of blood pressure. Genes such as *MAPK3*, *NOS3*, *AGTR1*, *DECR1*, *VEGFA*, *GNB3*, *APOE*, *CYP11B2*, *EGFR*, *POMC*, and *STAT3* were highly interactive (Figure 7). Some of these genes are a part of the AGE/RAGE pathway (*NOS3*, *MAPK3*, *STAT3*, and *EGFR*). Several studies have shown a link between *AGE–RAGE* stress and the pathophysiology of a variety of cardiovascular disorders, including essential and pulmonary hypertension [75]. On the other hand, the *AGTR1* and *CYP11B2* genes are linked to the ACE inhibitor pathway and are used to treat cardiovascular disorders such as hypertension [76]. ACE inhibitors prevent ACE from transforming angiotensin I into angiotensin II, lowering blood pressure by suppressing smooth muscle constriction and decreasing aldosterone release [77]. Additionally, *GNB3*, *VEGFA*, and *APOE* genes function on the cell-size level, where they are mostly linked to artery morphogenesis [78,79].

### 4.3. Differences and Similarities between the GWAS and Text Mining

Combining text mining and GWAS analysis yielded a set of genes that were shared by both methods of analysis (Figure 10 and Supplementary S8). Most of these genes are correlated with heart muscle activity and heart rate and are associated with cell proliferation and cell migration (*HDAC4*, *EPAS1*, *SCN5*, *FGFR3*, *AKT3*, and *ULK4*). For instance, histone deacetylases (HDACs) and histone acetyltransferases (HATs) regulate a broad range of biological processes by controlling transcription factors’ accessibility to the gene promoter by histone acetylation or deacetylation [80]. *HDAC4* is one of these genes that has been connected to cardiovascular disorders, where in cultured cardiomyocytes, it regulates neointimal hyperplasia by inducing the proliferation and migration of vascular smooth muscle cells [81]. *CYP11B2* is one of the most important genes that regulate hypertension manifestation due to its role as a key enzyme in aldosterone biosynthesis and aldosterone synthase. because the renin-angiotensin system is critical in regulating intravascular volume and blood pressure. The text mining analysis highlighted the importance of this gene by reflecting its high frequency of EH articles (Figure 7) and high interaction activity in PPI analysis (Figure 7). The GWAS reported no SNPs near or close to this or similar genes; this could be due to the exclusion criteria used to recruit the Qatari cases for EH, which excluded cases with secondary causes of EH such as kidney disease and diabetes. We excluded these individuals to focus our search on just the genes that are associated with only the EH phenotype without interference from secondary causes.

### 4.4. Allele Frequency of EH-Associated SNPs across Ethnic Groups

Additionally, we studied the allele frequency of EH-associated SNPs across a range of ethnic groups (Figure 11 and Supplementary S9). The clustering analysis of SNPs’ frequency across different ethnic groups shows a high frequency for the minor allele, especially in AFR, EAS, and SAS populations, which are more likely to be risk alleles [82] (Figure 11 and Supplementary S9). The AFR population was distinguished from the other populations, whereas SAS and EAS were grouped together. According to the World Health Organization (WHO), hypertension affects approximately 46% of adults aged 25 and older in Africa, compared to 35 to 40% in other parts of the world [83]. This could explain the high divergence of the AFR population in the EH-associated SNP data compared to other ethnic groups (Supplementary S5). The majority of the EH-associated SNPs that differentiated between ethnic groups are located near the *SPATS2L* gene, which has previously been linked to blood pressure in some ethnic groups such as Koreans [84,85].

## 5. Conclusions

We combined a GWAS for 100 hypertensive patients and 100 normal controls and text mining of over 10,000 research articles relevant to EH-gene association to map unique genes/variants to our patients with hypertension; we mapped 194 unique to our Qatari patients that are significantly associated with hypertension. Of these, 26 SNPs in 13 genes have a log-*p* value ≥ 4. The most significant region was the intergenic region of *C2orf47-SPATS2L*, which contains nine EH-associated SNPs that are highly related to hypertension, which was not previously reported in the literature. Interestingly, we identified three novel SNPs, rs_new-95 (4.70 log-*p* value), rs_new-58, and rs_new-56, located in the loci *RBM47*, *TMEFF2-AC013401.1*, and AC067956.1, respectively. The gene–disease association analysis identified 60 EH-associated genes that are linked to 270 different cardiovascular diseases. The *SCN5A* gene that showed a correlation between rs74947646 and EH with a log-*p* value of 3 is linked to more than 121 cardiovascular disorders. The *RYR2* gene was linked to 60 different cardiovascular diseases, the majority of which affect the functionality of ventricular hypertrophy. Our research found that rs1391189881, which is located in *RYR2*, has a significant association with EH, with a log-*p* value of 3. Furthermore, genes of *SCN10A*, *EPAS1*, *NPPC*, *ACKR3*, *CHRM3*, and *HDAC4* were linked to 51, 28, 27, 27, 23, and 23 cardio disorders, respectively. In addition, we identified 21 common genes that are shared between the GWAS and text mining analyses; of these, *FNDC3B*, *FHIT*, *NPPC*-*DIS3L2*, *GLI2*, and *RBM47* genes are the most highly associated with hypertension. Successfully, our analysis revealed two SNPs with a hypertension association of ≥5 *p* value log-*p* value (rs921932379 and rs113688672), which are located within the intergenic region of *GMPS-SETP14* and *ISCA1P6-AC012451.1* genes. Allele frequency of our GWAS indicates that our patients’ susceptibility to hypertension are more related to Africans and Asians than other ethnic groups. Text mining analysis aided GWAS analysis by providing a comprehensive catalogue of known and previously undiscovered EH-associated genes. The limitation of our study is the low number of WGS samples; therefore, more patients with EH need to be included to increase the statistical power of association with hypertension in our population.

## Figures and Tables

**Table 1:** Top SNP markers associated with EH in studied Qatari population (*p* ≤ 1 × 10^−^^5^).

SNP ID	Chr	Position	Ref	Alt	Gene	AC	FET Logpval
rs921932379	3	155674348	T	TA	GMPS-SETP14	17	5.27
rs113688672	2	129289164	C	T	ISCA1P6-AC012451.1	16	4.95
rs_new-95	4	40521676	CTTTTTTTTTTTTT	C	RBM47	16	4.7
rs35166853	2	232850730	C	T	DIS3L2	15	4.63
rs11591086	1	30925979	G	A	RP4-591L5.2-MATN1	14	4.32
rs11893181	2	18313644	G	A	KCNS3	14	4.32
rs1491266756	2	52292323	TTA	T	AC007682.1	14	4.32
rs17199733	2	232824585	G	A	DIS3L2	14	4.32
rs34189801	2	232808692	G	A	NPPC-DIS3L2	14	4.32
rs34553499	2	232820162	C	T	NPPC-DIS3L2	14	4.32
rs60554757	3	122342303	G	A	PARP15	14	4.32
rs652625	1	12225351	T	A	TNFRSF1B	14	4.32
rs66825295	2	216896216	GAGAA	G	MREG	14	4.32
rs6752063	2	121556047	G	A	GLI2	14	4.32
rs73858324	3	122337678	G	A	PARP15	14	4.32
rs61762198	1	3500434	G	GCAGCCACCAGACAACGCA	MEGF6	17	4.28
rs1217727360	2	230419573	A	AAAGAG	DNER	16	4.25
rs369201055	2	138779802	A	AAC	HNMT-AC069394.1	15	4.19
rs_new-56	2	193355414	A	ACAGGGCTGCAGGAAAAAGGGAATGCCTATAGAC	TMEFF2-AC013401.1	8	4.11
rs1345206935	3	18848380	G	GTTTTTTTTTTTTTTTTTTTTTTTTTT	AC144521.1	13	4.09
rs57679512	3	61057088	GA	G	FHIT	13	4.03
rs7646137	3	162408311	T	A	RP13-526J3.1-RP11-10O22.1	15	4.01
rs770631619	1	72759661	C	CTTTTTTTTTTTTT	NEGR1-RPL31P12	15	4.01
rs10931882	2	200917613	T	C	C2orf47-SPATS2L	13	4
rs10931883	2	200917675	T	C	C2orf47-SPATS2L	13	4
rs11291368	3	172047831	AG	A	FNDC3B	13	4
rs11897782	2	200916495	G	A	C2orf47-SPATS2L	13	4
rs11903185	2	200916506	T	C	C2orf47-SPATS2L	13	4
rs12032899	1	3999592	G	C	RP13-614K11.1	13	4
rs12464998	2	200893820	T	C	C2orf47-SPATS2L	13	4
rs12470336	2	201000860	A	T	C2orf47-SPATS2L	13	4
rs2700337	1	96116213	A	G	RP11-286B14.1	13	4
rs4673652	2	200903578	C	T	C2orf47-SPATS2L	13	4
rs73141887	2	8590888	T	G	LINC00299-AC011747.3	13	4
rs73858322	3	122335609	T	A	PARP15	13	4
rs7608345	2	8590943	G	A	LINC00299-AC011747.3	13	4
rs77321003	2	201005171	C	A	C2orf47-SPATS2L	13	4
rs79953652	2	200913138	T	C	C2orf47-SPATS2L	13	4

## Data Availability

Data available within the article or its supplementary materials.

## Supplementary Materials

The following supporting information can be downloaded at: https://www.mdpi.com/article/10.3390/jpm12050722/s1. Supplementary S1: A summary table comparing the values of control and patient clinical parameters. Supplementary S2: Computer programming scripts used for text mining; Supplementary S3: Genes studied according to their number of EH-associated SNPs; Supplementary S4: Fisher’s exact test scores for SNPs’ association with EH; Supplementary S5: Genes containing EH-associated SNPs; Supplementary S6: The cardiovascular disorders related to EH-associated SNPs detected in the current study; Supplementary S7: The highest frequently mentioned genes and SNPs in the hypertension literature; Supplementary S8: Text mining and GWAS comparison using Venn analysis; Supplementary S9: The allele frequency of EH-associated SNPs in different ethnic groups according to Ensemble database.
