# Supplementary material for: Whole-Genome Sequencing of 100 Genomes Identifies a Distinctive Genetic Susceptibility Profile of Qatari Patients with Hypertension
Source: J Pers Med. 2022 Apr 29;12(5):722. doi: 10.3390/jpm12050722 (PMC9144388; doi:10.3390/jpm12050722)
Supplement: Supplementary file 1 [file jpm-12-00722-s001.zip › File_S2.pdf]

```
---
output:
  pdf_document: default
  html_document: default
---
#Do Correlation analysis for genes
library(corrplot)
library(RColorBrewer)
library(tidyverse)
library(corrr)
library(packcircles)
library(ggplot2)



######################### Data Analysis
#read gene file
genedata=read.table("Gene_Frequency.tsv", header = TRUE,row.names = 1)


################ Create bubbles
colSums(genedata)
genefreq <- data.frame(genename = names(genedata),freq=colSums(genedata))
write.table(genefreq,"gene_frequency_table.tsv",sep = "\t")

packing <- circleProgressiveLayout(genefreq$freq, sizetype='area')
data <- cbind(genefreq, packing)
dat.gg <- circleLayoutVertices(packing, npoints=50)

pdf("circle_freq_plot.pdf")
# Make the plot
ggplot() +
  # Make the bubbles
  geom_polygon(data = dat.gg, aes(x, y, group = id, fill=as.factor(id)), colour = "black", alpha = 0.6)
+
  # Add text in the center of each bubble + control its size
  geom_text(data = data, aes(x, y, size=freq, label = genename)) +
  scale_size_continuous(range = c(1,4)) +
  # General theme:
  theme_void() +
  theme(legend.position="none") +
  coord_equal()
dev.off()


#########################Correlation
# mat : is a matrix of data
# ... : further arguments to pass to the native R cor.test function
#cor.mtest <- function(mat, ...) {
#  mat <- as.matrix(mat)
#  n <- ncol(mat)
#  p.mat<- matrix(NA, n, n)
```

```
#  diag(p.mat) <- 0
#  for (i in 1:(n - 1)) {
#    for (j in (i + 1):n) {
#      tmp <- cor.test(mat[, i], mat[, j], ...)
#      p.mat[i, j] <- p.mat[j, i] <- tmp$p.value
#    }
#  }
#  colnames(p.mat) <- rownames(p.mat) <- colnames(mat)
#  p.mat
#}

#p.mat <- cor.mtest(genedata)
#M<-cor(genedata)
#for(i in 1:nrow(M)){
#  for(j in 1:nrow(M)){
#    if(p.mat[i,j] > 0.001){
#      M[i,j] <- 0
#    }
#  }
#}

#pdf("Gene_Network.pdf")
#network_plot(M,min_cor = 0.3)
#dev.off()
#
#pdf("CorrPlot.pdf")
#corrplot(M, type="upper", order="hclust",
#         col=brewer.pal(n=8, name="RdYlBu"))
#dev.off()

import sys
import re
import pandas as pd
import json



def remove_punctuations(my_str):
	punctuations = '''!()-[]{};:'"\,<>./?@#$%^&*_~、|'''
	no_punct = ""
	for char in my_str:
		if char not in punctuations :
			no_punct = no_punct + char
		else:
			no_punct = no_punct + " "
	return no_punct

def count(abstr,HuGenes):
	genescount={}
	uniq={}
	abstr=abstr.split(" ")
	for l in abstr:
		uniq[l]=1
```

```
	for k in uniq.keys():
		if len(k) >= 3 and k not in ("not"):
			if k in HuGenes.keys():
				genescount[HuGenes[k]]=1
	return list(genescount.keys())

def read_gene_file(filepath):
	HuGenes = {} #human genes
	with open(filepath, 'r') as fp:
	   HuGenes = json.load(fp)
	return HuGenes

def read_pubmed(filepath):
	pubmedabs={}
	abstract=""
	with open(filepath,"r") as myfile:
		for line in myfile:
			abstract+=line.strip()+" "
			if "PMID:" in line:
				pubmid=re.findall("PMID: (\d+)",line)[0].replace("PMID: ","")
				abstract=remove_punctuations(abstract)
				pubmedabs[pubmid]=abstract
				abstract=""
	return pubmedabs



pubmedabs  = read_pubmed(sys.argv[1])
Humangenes = read_gene_file("symbol-map.json")
genesymbole= read_gene_file("id_gene_symbole.json")

#create pubmed jason file
pubmedwithgenes={}
for p in pubmedabs:
	genes=count(pubmedabs[p],Humangenes)
	if len(genes) == 0:
		continue
	pubmedwithgenes[p]=genes
#free memory
pubmedabs={}
Humangenes={}

########################### Filter genes Only More Than 30 article
allgenes={} #all genes
for pid in pubmedwithgenes:
	for gene in pubmedwithgenes[pid]:
		if gene not in allgenes:
			allgenes[gene]=1
		else:
			allgenes[gene]+=1
freqgenes={}
for gene in allgenes:
```

```
    if allgenes[gene] >=30:
        freqgenes[gene]=allgenes[gene]
allgenes=freqgenes
freqgenes={}
##########################


allgenes=list(allgenes.keys())
allpid=list(pubmedwithgenes.keys())


print("gene",end="\t")
print('\t'.join(genesymbole[str(x)] for x in allgenes))
for pd in allpid:
    print(pd,end="\t")
    pid_genes={}
    for g in pubmedwithgenes[pd]:
        pid_genes[g]=1
    for gene in allgenes:
        if gene in pid_genes:
            print("1",end="\t")
        else:
            print("0",end="\t")
    print()
```

```
python3 pubmed-count.py  > Gene_Frequency.tsv
Rscript Plot_By_R.r
 esearch -db pubmed -query "hypertension + gene" | efetch -format abstract >
hypertension_PUBS.txt
 1059  esearch -db pubmed -query "obesity + gene" | efetch -format abstract > obesity_PUBS.txt
 1062  esearch -db pubmed -query "diabetes + gene" | efetch -format abstract > diabetes_PUBS.txt
```
