# Supplementary figures and images for: Whole-Genome Sequencing of 100 Genomes Identifies a Distinctive Genetic Susceptibility Profile of Qatari Patients with Hypertension
Source: J Pers Med. 2022 Apr 29;12(5):722. doi: 10.3390/jpm12050722 (PMC9144388; doi:10.3390/jpm12050722)

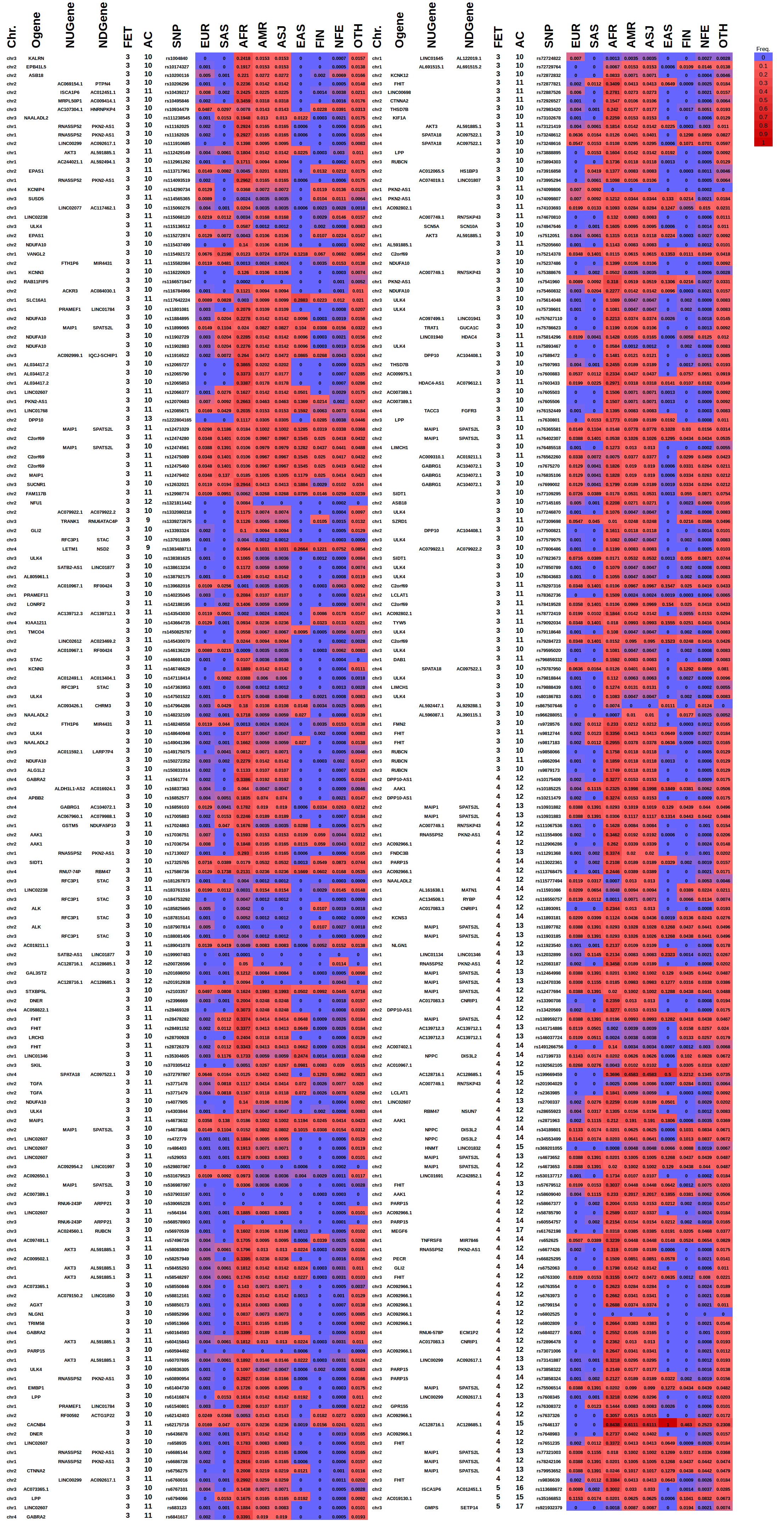

Supplement: Supplementary file 1 [file jpm-12-00722-s001.zip › File_S9.jpg]
